# Impact of replacing sedentary behaviour with other movement behaviours on depression and anxiety symptoms: a prospective cohort study in the UK Biobank

**DOI:** 10.1186/s12916-021-02007-3

**Published:** 2021-06-17

**Authors:** A. A. Kandola, B. del Pozo Cruz, D. P. J. Osborn, B. Stubbs, K. W. Choi, J. F. Hayes

**Affiliations:** 1 Division of Psychiatry, University College London, Maple House, 149 Tottenham Court Rd, London, W1T 7BN UK; 2 Institute of Mental Health, University College London, London, UK; 3 Motivation and Behaviour Program, Institute for Positive Psychology and Education, Faculty of Health Sciences, Australian Catholic University, Sydney, Australia; 4 Camden and Islington NHS Foundation Trust, London, UK; 5 Department of Psychological Medicine, Institute of Psychiatry, Psychology, and Neuroscience, King’s College London, London, UK; 6 Physiotherapy Department, South London, and Maudsley National Health Services Foundation Trust, London, UK; 7 Department of Psychiatry, Massachusetts General Hospital, Boston, MA USA; 8 Department of Epidemiology, Harvard T.H. Chan School of Public Health, Boston, MA USA; 9 Psychiatric & Neurodevelopmental Genetics Unit, Center for Genomic Medicine, Massachusetts General Hospital, Boston, MA USA; 10 Stanley Center for Psychiatric Research, Broad Institute, Boston, MA USA

**Keywords:** Sedentary behaviour, Depression, Anxiety, Compositional, Physical activity, MVPA

## Abstract

**Background:**

Sedentary behaviour is potentially a modifiable risk factor for depression and anxiety disorders, but findings have been inconsistent. To assess the associations of sedentary behaviour with depression and anxiety symptoms and estimate the impact of replacing daily time spent in sedentary behaviours with sleep, light, or moderate to vigorous physical activity, using compositional data analysis methods.

**Methods:**

We conducted a prospective cohort study in 60,235 UK Biobank participants (mean age: 56; 56% female). Exposure was baseline daily movement behaviours (accelerometer-assessed sedentary behaviour and physical activity, and self-reported total sleep). Outcomes were depression and anxiety symptoms (Patient Health Questionnaire-9 and Generalised Anxiety Disorders-7) at follow-up.

**Results:**

Replacing 60 min of sedentary behaviour with light activity, moderate-to-vigorous activity, and sleep was associated with lower depression symptom scores by 1.3% (95% CI, 0.4–2.1%), 12.5% (95% CI, 11.4–13.5%), and 7.6% (95% CI, 6.9–8.4%), and lower odds of possible depression by 0.95 (95% CI, 0.94–0.96), 0.75 (95% CI, 0.74–0.76), and 0.90 (95% CI, 0.90–0.91) at follow-up.

Replacing 60 min of sedentary behaviour with moderate-to-vigorous activity and sleep was associated with lower anxiety symptom scores by 6.6% (95% CI, 5.5–7.6%) and 4.5% (95% CI, 3.7–5.2%), and lower odds of meeting the threshold for a possible anxiety disorder by 0.90 (95% CI, 0.89–0.90) and 0.97 (95%CI, 0.96–0.97) at follow-up. However, replacing 60 min of sedentary behaviour with light activity was associated with higher anxiety symptom scores by 4.5% (95% CI, 3.7–5.3%) and higher odds of a possible anxiety disorder by 1.07 (95% CI, 1.06–1.08).

**Conclusions:**

Sedentary behaviour is a risk factor for increased depression and anxiety symptoms in adults. Replacing sedentary behaviour with moderate-to-vigorous activity may reduce mental health risks, but more work is necessary to clarify the role of light activity.

**Supplementary Information:**

The online version contains supplementary material available at 10.1186/s12916-021-02007-3.

## Background

There are currently more than 320 million people living with depression and over 260 million with anxiety disorders worldwide [1]. Depression and anxiety disorders respectively account for the first and sixth most years lost to disability globally [1]. They are associated with elevated physical health risks, premature mortality, and substantial individual, social, and financial burden [2–5]. Physical activity and sedentary behaviour appear to be associated with the risk of depression and anxiety disorders and are potentially modifiable through targeted interventions [6–8].

Sedentary behaviour refers to any waking activity while sitting, reclining, or lying with low energy expenditure (≤ 1.5 metabolic equivalents), such as sitting while watching television or using a computer [9]. Sedentary behaviour levels have risen in recent years [10–12], typically accounting for 60% of waking time in adults [13]. Sedentary behaviour is gaining recognition as a risk factor for several long-term conditions independent of physical activity [14–16]. For example, meeting nationally recommended moderate-to-vigorous intensity physical activity guidelines per week do not necessarily ameliorate the health risks of high sedentary behaviour [17, 18].

There is some evidence that high sedentary behaviour is an independent risk factor for depression and anxiety disorders in adults, but findings have been inconsistent [7, 8, 19–21]. Reducing sedentary behaviour is possible through increasing physical activity, which itself is associated with a lower incidence of depression and anxiety disorders [6, 22, 23] and can reduce depression and anxiety symptoms in those with a disorder [24–27]. However, most studies focus on moderate-to-vigorous forms of activity that typically account for around 4% of waking time in adults [13], such as running or cycling. If sedentary behaviour is an independent risk factor for depression and anxiety symptoms, more substantial changes to daily movement patterns may be necessary to mitigate these risks, such as increasing in light-intensity activity.

Inconsistent findings for sedentary behaviour as risk factor for depression and anxiety symptoms could be due to methodological limitations in previous work, including the cross-sectional nature of most studies, use of self-report measures of activity that induce substantial measurement error [28, 29], and focus only on depression outcomes, despite the substantial global burden of anxiety disorders [1] and their co-morbidity with depression [30]. Previous studies have also been unable to appropriately account for the co-dependence of sedentary behaviour and physical activity [31]. As time is finite in a day, time spent in sedentary behaviour will necessarily displace time in at least one other behaviour, such as physical activity or sleep. These other behaviours may also have their own positive or negative effects on mental health that we must consider when estimating the effect of reducing sedentary behaviour. Traditional analytical methods assume these are independent, such that increasing sedentary behaviour time will not influence physical activity or sleep time [31].

Advances in sedentary behaviour and physical activity research will require the use of novel methodologies for examining movement behaviours within a 24-h cycle, including sleep [32]. Compositional data analysis is a set of statistical principles and techniques for handling data representing proportions of a finite whole, such as periods of different activities within a day, recently applied to physical activity data [31, 33]. The method allows the assessment of associations between sedentary behaviour time and depression and anxiety disorders while accounting for all other periods of the day (e.g. physical activity and sleep), and also to estimate the potential impact of replacing sedentary behaviour with other activities.

Only three studies have used composition methods with mental health outcomes, each using cross-sectional designs in small samples [34–36]. These studies suggest that replacing sedentary behaviour with activity of different intensities could lower mental health risks. Associations between time in sedentary behaviour and mental health may depend on how time in the rest of the day is structured [35]. Not accounting for this could have contributed to previous studies’ inconsistent findings without compositional methods [7, 8, 19–21]. For example, a recent systematic review of 12 prospective studies found that sedentary behaviour was associated with a higher risk of depression, but this was attenuated when adjusting for physical activity [7]. Estimating the potential impact of replacing time in sedentary behaviour for activity of different intensities or sleep on the risk of depression and anxiety disorders would also directly impact public health policy. For example, it would determine the types of activities to promote to replace sedentary behaviour.

There is a lack of prospective studies that use compositional methods to assess mental health outcomes or examine associations between device-measured sedentary behaviour and depression and anxiety disorders. Compositional methods are most suited to analysing data from devices that continuously capture the full spectrum of activity intensities over 24 h. The self-report measures of activity in prior prospective studies are less reliable than devices for estimating sedentary time [37, 38] and tend to poorly estimate light-intensity activity, which accounts for most daily movement [39]. To address previous limitations in the field, we conducted a prospective cohort study to (1) determine how accelerometer-derived sedentary behaviour is associated with depression and anxiety symptoms while accounting for physical activity and sleep in a 24-h period and (2) estimate the effect of replacing daily sedentary time with other movement behaviours (sleep, light, and moderate-to-vigorous activity) on the depression and anxiety symptoms.

## Methods

### Participants

We used data from the UK Biobank, a prospective cohort study of 502,682 participants (5.5% response rate) aged 40 to 69 years recruited from the general population of England, Scotland, and Wales, between April 2006 and December 2010 [40]. The Biobank study recruited participants in accordance with its aims of assessing disease in middle-to-older aged adults, with sample sizes based on statistical power calculations showing that at least 5000 to 10,000 cases of any condition would be necessary to reliably detect exposure-outcome associations with 1.3 to 1.5 odds ratios and around 20,000 for associations with at least 2.0 odds ratios [41]. At baseline, participants completed various questionnaires, physical measures, imaging, genetic, and biological assessments in 22 research centres across the UK [42]. Participants who provided a valid e-mail address at baseline, 236,507 (47.1%) were invited to wear an accelerometer for seven days between February 2013 and December 2015. Researchers chose participant e-mail addresses at random, except for those in the North West region, to avoid overburdening participants who had already been recruited into trials for other new projects. A total of 103,706 participants (20.6%) agreed to wear the accelerometer, and 99,608 provided sufficient quality data for analysis [43]. Our study includes a complete case analysis of participants with full accelerometer (exposure) and covariate data at baseline and Patient Health Questionnaire-9 (PHQ-9) and Generalised Anxiety Disorder-7 (GAD-7) (outcomes) at follow-up in 2017 (n = 60,235). A flowchart of participants in this study is available in the Supplementary materials (Figure 1 of the Supplementary Materials).

### Outcomes: depression and anxiety symptoms

Depression and anxiety symptoms were measured at follow-up using full PHQ-9 and GAD-7 scales. The PHQ-9 is a well-validated, 9-item screening tool for depressive symptoms [44], with scores ranging from 0 to 27. The GAD-7 is a 7-item scale that is a validated screening tool for symptoms of generalised anxiety disorders, with scores ranging from 0 to 21 [45]. For both scales, we used continuous symptom scores as our primary outcome more closely represent the reality of how mental health symptoms manifest on a continuum and to maximise statistical power [46]. We also estimated possible cases of depression and anxiety disorders at follow-up using established cut-off scores (scores ≥ 10) [44, 45].

### Exposures: daily movement behaviours

Daily movement behaviours were categorised as sedentary behaviour, light activity, moderate-to-vigorous activity, and sleep. Axivity AX3 triaxial accelerometers were worn on the wrist to estimate physical activity and sedentary behaviour. We describe the protocols for collecting and processing this data in the Supplementary Materials (Methods 1). We followed protocols of previous studies [47–49] to define sedentary behaviour, light, and moderate-to-vigorous activity over 5-s epochs as averaged Euclidean Norm Minus One values of ≤ 30 milli-*g* (minus self-reported sleep duration), > 30 milli-*g* and < 125 milli-g and ≥ 125 milli-*g*, respectively. We calculated the average daily time at each intensity across the recording period per participant. We derived the sleep duration variable from a touchscreen questionnaire that participants completed at baseline. The questionnaire asked: “About how many hours sleep do you get in every 24 hours? (please include naps)”. The questionnaire automatically rejected responses of less than 1 or over 23 and asked participants to confirm responses of < 3 or > 12. We subtracted total daily sleep time from the daily sedentary behaviour, which was included in the model with light activity, and moderate-to-vigorous activity time to make up the full 24 h.

### Covariates

We selected possible confounding variables based on our understanding of the possible causal structure of our proposed exposure-outcome association from discussions between co-authors and previous literature [50]. We used directed acyclic graphs (DAGs) to outline causal associations between movement behaviours, depression and anxiety symptoms, and possible confounding variables (see Figure 2 of the Supplementary Materials). Using the DAG, we identified several confounding variables that require statistical adjustment in our models to block backdoor exposure-outcome pathways and more closely approximate a direct effect of movement on depression and anxiety symptoms (for more information on structural definitions of confounding, see [51]). The confounding variables for this analysis included: age, sex, socioeconomic position (household income of < £18,000, £18,000 to £30,999, £31,000 to £51,999, £52,000 to £100,000, and > £100,0000), smoking status (current, former, or never), baseline depression and anxiety symptom scores, education (degree, A/AS-level, O-level/GCSE, CSE, NVQ/HND/HNC, other qualifications, none), chronic illness (self-reported yes or no), and diet (portions of fruit and vegetables per day). We did not adjust for body mass index as it may be on the causal pathway between movement and depressive and anxiety symptoms.

Baseline depression and anxiety were measured with a short version of the PHQ-9, a PHQ-4 [44]. Three questions covered core features of depression (low mood, anhedonia, and lethargy), and the fourth was adapted to measure tenseness, a common feature of anxiety disorders. Participants responded on a four-point Likert scale from 0 (not at all) to 3 (nearly every day). Scores ranged from 0 to 12, in which higher scores indicated more severe symptoms. Ultra-brief adaptations of the PHQ-9 have good agreement with full scales of depression and anxiety symptoms [52]. In a representative, non-clinical sample of middle-aged adults (n = 5003), the PHQ-4 showed good internal consistency (Cronbach’s alpha = 0.82), factorial and structural validity. Our a priori DAGs suggested that adjustment for these variables would be necessary to estimate causal associations between movement behaviours on depression and anxiety symptoms.

### Analysis

We reported all descriptive variables using arithmetic means and standard deviations for normal distributions and medians and interquartile ranges for non-normal distributions. We also used geometric means to describe the daily physical activity, sedentary, and sleep time, which is a measure of central tendency that accounts for the compositional nature of the variables [53].

The main analysis used compositional data analysis (described below) to examine associations between sedentary behaviour and depression and anxiety symptoms scores while accounting for physical activity and sleep (aim 1). Within this analysis, we estimate how replacing sedentary time with sleep, light, or moderate-to-vigorous activity affects future depression and anxiety symptoms scores (aim 2).

### Main analysis

Detailed descriptions of compositional analysis approaches to physical activity data are available elsewhere [33], and we provide a brief description in the Supplementary Materials (Methods 2).

Each exposure of interest in our analysis is a composition of average daily sleep, sedentary behaviour, light, and moderate-to-vigorous activity across the recording period, which we normalised to the proportion of 1440 min [33], the total time in a day. We used a pivot coordinate approach whereby we calculated a set of three isometric log-ratio coordinates per participant that represents their total relative time in each movement behaviour per day. The first (pivot) coordinate represents daily sedentary relative to the geometric mean of all other daily movement behaviours, i.e., sleep, light, and moderate-to-vigorous activity:

$$ \sqrt{\frac{3}{4}}\ln \frac{\mathrm{sedentary}\ \mathrm{behaviour}}{\left(\mathrm{sleep}.\mathrm{light}.\mathrm{moderate}\ \mathrm{to}\ \mathrm{vigorous}\ \mathrm{activity}\right)} $$

The other two log-ratio coordinates contain relative information representing the remaining time in a participant's total daily composition, i.e., sleep over light and moderate-to-vigorous and light over moderate-to-vigorous activity. We entered these sets of coordinates as our exposure variables into the regression models with either one of the two primary outcomes (depression or anxiety). This provides a base model that allowed us to assess the overall associations of sedentary behaviour with depression and anxiety symptoms scores while accounting for all other movement behaviours in the day (aim 1). We used negative binomial regressions for these base models due to the right skew distribution and over-dispersion of the mental health outcomes (see Figures 3 and 4 of the Supplementary Materials).

To then estimate the effect of replacing sedentary behaviour with other behaviours on depression and anxiety symptom scores (aim 2), we used a change-matrix approach described in detail elsewhere [33]. The base model’s coefficients represent the estimated effect on depression and anxiety symptom scores when sedentary behaviour (numerator) changes relative to the geometric mean of all other time-use variables (denominator). The change-matrix procedure uses these base model coefficients to simulate different scenarios, such that we can theoretically reduce time in sedentary behaviour and increase time in either sleep, light, or moderate-to-vigorous activity isometrically to estimate the possible effect on depression or anxiety. We use the term theoretical replacements as the estimates are based on simulations rather than actual changes in movement behaviours in the data. We examined how theoretically replacing 1 to 60 min of sedentary behaviour with the other behaviours was associated with depression and anxiety symptom scores, using coefficients from the base model. We estimated replacements up to 60 min to align with previous studies using compositional methods with mental health outcomes [35]. It is possible to estimate replacements of up to 1440 min, but substantial reductions in daily sedentary behaviour are less plausible in the population.

To aid interpretation of the final models, we back-transformed all log-ratio coordinates into the original units so that model coefficients represent changes in minutes per day of each movement behaviour. We presented the outputs of all negative binomial regression models as percentage changes in PHQ-9 and GAD-7 scores. We also ran logistic models using the same exposure and confounding variables, with the dichotomized outcome variables indicating new cases of either depression or anxiety (a score of ≥ 10 on the PHQ-9 or GAD-7). Fully-adjusted models included all confounding variables that we describe above.

### Sensitivity analysis

We ran sensitivity analyses to test the robustness of our findings and alternative explanations. We repeated the main analysis and excluded all participants with a self-reported history of depression or anxiety to further reduce the risk of reverse causation. To estimate the plausibility of bias from unmeasured and residual confounding, we also calculated e-values for our main findings [54]. The e-value estimates the strength of an unmeasured confounding variable would require to nullify the observed associations between our exposure and outcomes while accounting for all measured covariates [55].

All analyses were conducted in Stata 15 and R (version 4.0.0) using the Compositions [56] and zCompositions [57] packages.

## Results

### Participants

The sample included 60,235 participants with complete exposure, outcome, and covariate data in the main analysis. Around 3774 (6.2%) participants met the PHQ-9 threshold for possible depression and 2216 (3.7%) for the GAD-7 threshold for possible anxiety, and 4096 (6.8%) met the criteria for both at follow-up, 2 years after baseline. Table 1 contains baseline characteristics for our subsample of participants (n = 60,235) and the remaining UK Biobank sample (n = 442,587).

**Table 1:** Baseline characteristics for included and remaining UK Biobank sample

Characteristic	Included (n = 60,235)	Remaining sample (n = 442,278)
Age, mean (SD)	55.9 (7.7)	56.6 (8.1)
Sex		
Female	33,739 (56%)	239,649 (54%)
Male	26,496 (44%)	202,629 (46%)
Ethnicity		
White	58,649 (98%)	414,054 (94%)
Mixed	282 (0.5%)	2676 (0.6%)
South Asian	409 (0.7%)	9473 (2.2%)
Black	373 (0.6%)	7688 (1.7%)
Chinese	108 (0.2%)	1466 (0.3%)
Other	281 (0.5%)	4277 (1.0%)
Household income		
Less than 18,000	8102 (13%)	89,100 (24%)
18,000 to 30,999	14,200 (24%)	93,977 (26%)
31,000 to 51,999	17,402 (29%)	93,371 (26%)
52,000 to 100,000	15,745 (26%)	70,522 (19%)
Greater than 100,000	4786 (7.9%)	18,144 (5.0%)
Education		
College or University degree	28,844 (48%)	132,321 (31%)
A levels/AS levels or equivalent	8237 (14%)	47,086 (11%)
O levels/GCSEs or equivalent	11,533 (19%)	93,666 (22%)
CSES or equivalent	2036 (3.4%)	24,851 (5.8%)
NVQ or HND or HNC or equivalent	2962 (4.9%)	29,767 (6.9%)
Other professional qualifications e.g., nursing, teaching	2950 (4.9%)	22,854 (5.3%)
None	3673 (6.1%)	81,600 (19%)
Smoking status		
Never	34,847 (58%)	238,678 (54%)
Previous	21,436 (36%)	151,624 (35%)
Current	3952 (6.6%)	49,027 (11%)
BMI, mean (SD)	26.6 (4.5)	27.6 (4.8)
Long-term physical illness		
Do not know	962 (1.6%)	10,425 (2.4%)
No	43,003 (71%)	286,253 (65%)
Yes	16,270 (27%)	143,630 (33%)
Diet	4.94 (2.48)	4.88 (2.80)
Baseline depression and anxiety symptoms, mean (SD)	1.34 (1.80)	1.64 (2.11)
Parental depression		
No	54,430 (90%)	383,357 (91%)
Yes	5805 (9.6%)	36,752 (8.7%)
Daily sedentary behaviour, arithmetic mean minutes (SD)	647.8 (99.2)	-
Daily light activity, mean arithmetic minutes (SD)	292.4 (62.7)	-
Daily moderate-to-vigorous arithmetic activity, mean minutes (SD)	65.1 (37.1)	-
Daily sleep, arithmetic mean minutes (SD)	434.8 (58.4)	-
Sedentary behaviour, geometric mean	0.45	-
Light activity, geometric mean	0.20	-
Moderate-to-vigorous activity, geometric mean	0.05	-
Sleep, geometric mean	0.30	-

*SD* standard deviation, *BMI* body mass index

### Main analysis

**Fig. 1:**
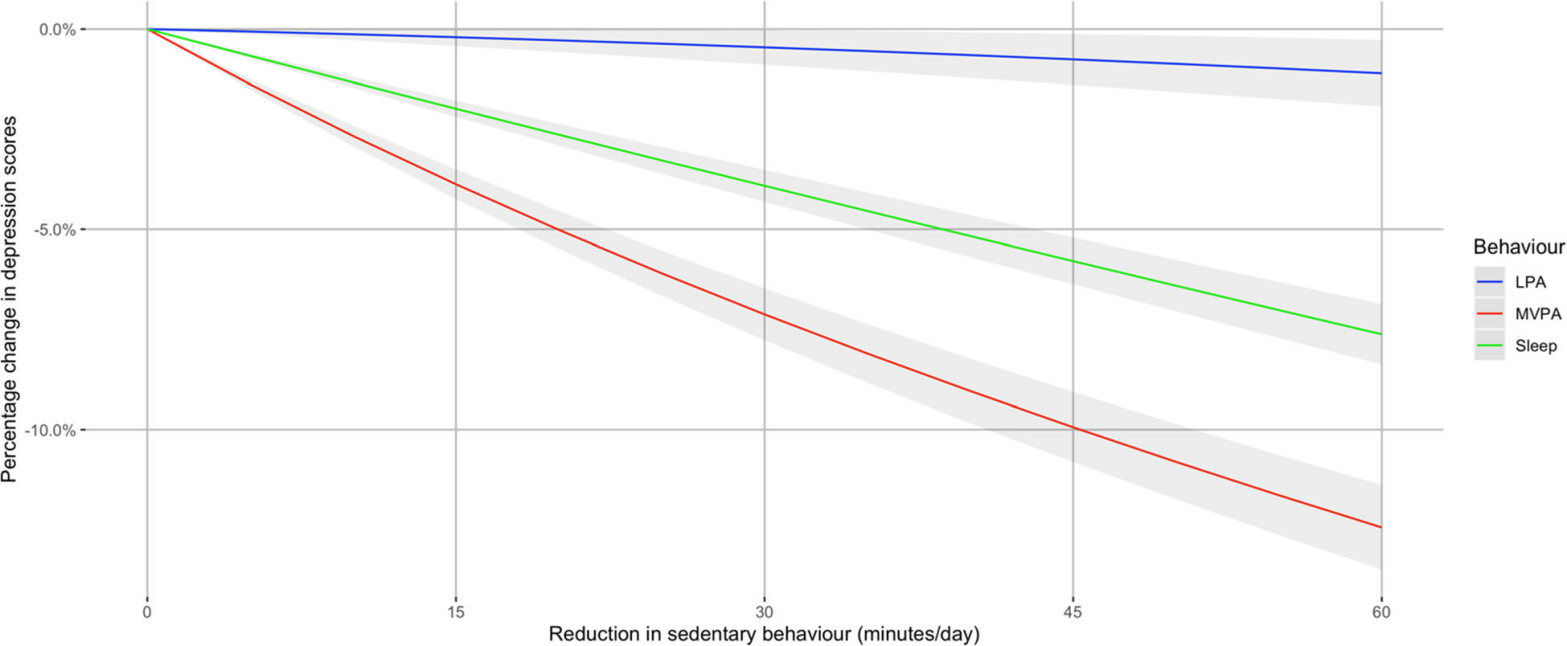
Effect of replacing daily sedentary time for other movement behaviours and sleep on depressive symptoms. LPA, light physical activity; MVPA, moderate-to-vigorous physical activity. Models are adjusted for age, sex, socioeconomic position, smoking, baseline depression and anxiety symptoms, education, chronic illness, and diet

In fully-adjusted base models, baseline time in sedentary behaviour was positively associated with depression (β = 0.49, 95% CI, 0.44–0.54, *p* < 0.001) and anxiety (β = 0.37, 95% CI, 0.31–0.44, *p* < 0.001) symptom scores at follow-up while accounting for time in light, moderate-to-vigorous activity, and sleep over 24 h (see Table 1 of the Supplementary Materials). Figure 1 shows the estimated effect on depressive symptom scores of potentially replacing sedentary behaviour with between 1 and 60 min of light, moderate-to-vigorous activity, or sleep. Replacing a total of 60 min of sedentary behaviour with 60 min of light activity, moderate-to-vigorous activity, and sleep in 24 h was associated with lower depression symptom scores by 1.3% (95% CI, 0.4–2.1%), 12.5% (95% CI, 11.4–13.5%), and 7.6% (95% CI, 6.9–8.4%), respectively.

In logistic models, potentially replacing 60 min of sedentary behaviour with 60 min of light, moderate-to-vigorous activity, and sleep in a 24-h period was associated with lower odds of possible depression, OR = 0.95 (95% CI, 0.94–0.96), OR = 0.75 (95% CI, 0.74–0.76), and OR = 0.90 (95% CI, 0.90–0.91), respectively.

**Fig. 2:**
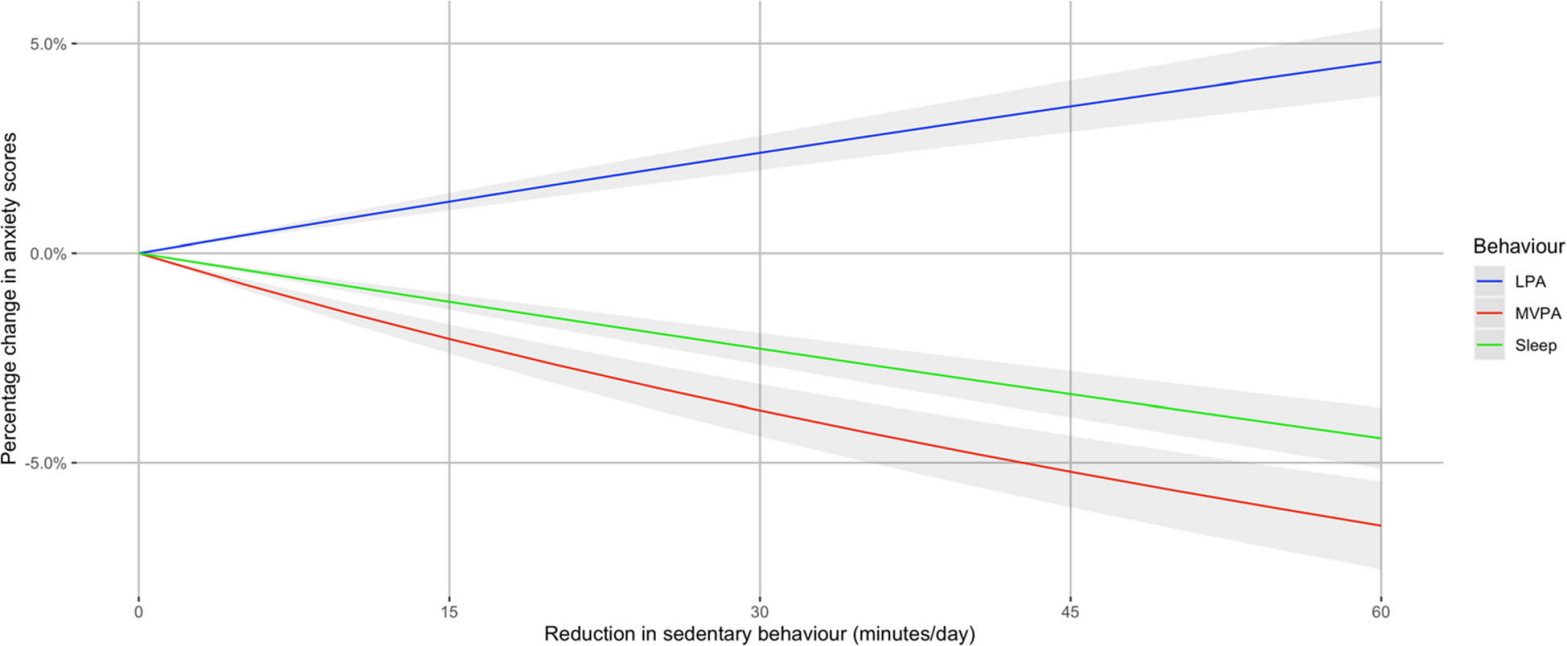
Effect of replacing daily sedentary time for other movement behaviours and sleep on anxiety symptoms. LPA, light physical activity; MVPA, moderate-to-vigorous physical activity. Models are adjusted for age, sex, socioeconomic position, smoking, baseline depression and anxiety symptoms, education, chronic illness, and diet

Figure 2 shows the estimated effect of replacing sedentary behaviour with activity or sleep on anxiety symptom scores. Replacing 60 min of sedentary behaviour with moderate-to-vigorous activity and sleep was associated with lower anxiety symptom scores by 6.6% (95% CI, 5.5–7.6%), and 4.5% (95% CI, 3.7–5.2%), while replacing with light activity was associated with higher anxiety symptom scores by 4.5% (95% CI, 3.7–5.3%).

In logistic models, replacing 60 min of sedentary behaviour with moderate-to-vigorous activity or sleep was associated with lower odds of possible anxiety by 0.90 (95% CI, 0.89–0.90) and 0.97 (95% CI, 0.96–0.97), while replacing with light activity was associated with higher odds by 1.07 (95% CI, 1.06–1.08).

### Sensitivity analysis

When we excluded participants with a history of depression or anxiety in our sample and reran the fully adjusted models in a sample of 39,973 (66% of our subsample) participants with complete data. The results were consistent with the findings of our main analysis and are presented in the Supplementary Materials (Results 1). The e-values indicate that our main findings are unlikely to be nullified by an unmeasured confounding variable and are presented in the Supplementary Materials (Results 2).

## Discussion

### Main findings

This study is the first to use compositional methods to examine prospective associations between 24-h movement behaviours and depression and anxiety symptoms in the population. We found that daily sedentary behaviour time at baseline was positively associated with depression and anxiety symptom scores at follow up. Theoretically replacing periods of daily sedentary behaviour with light, moderate-to-vigorous activity, or sleep at baseline was associated with lower depressive symptom scores at follow-up. Replacing sedentary behaviour with moderate-to-vigorous activity or sleep was also associated with lower anxiety symptom scores. We found that the most substantial estimated changes occurred when replacing sedentary behaviour with moderate-to-vigorous activity, where 60 min of replaced time resulted in 13% lower depression symptoms scores and 7% lower anxiety symptom scores.

Our findings indicate that sedentary behaviour is a possible risk factor for depression and anxiety disorders, which aligns with findings from several smaller studies that use self-report activity measures [7, 8, 19–21]. Recent meta-analyses have found no evidence of an association between sedentary behaviour and depression after adjusting for physical activity using standard methods (relative risk ratio = 1.03, 95% CI = 0.90–1.18, n = 4) [7]. However, we show that the association holds when using compositional methods to account for physical activity and sleep over 24 h.

We also modelled replacement effects to provide more realistic estimates of how reducing daily sedentary behaviour time might affect depression and anxiety symptom scores by considering possible replacements. For example, one recent meta-analysis found that high sedentary behaviour is associated with a 1.10 (95% CI, 1.03–1.19) higher risk of depression than low sedentary behaviour [7]. Another meta-analysis found that high total physical activity volume was associated with 0.83 (95% CI, 0.79–0.88) lower odds of depression than low total physical activity [6], whereas our results demonstrate the importance of considering how the time is replaced as reducing daily sedentary behaviour by an hour was associated with a larger effect size (OR = 0.75) when replaced with moderate-to-vigorous activity and smaller effect size (OR = 0.95) for light activity. These results also align with other studies indicating a potentially beneficial impact of light activity on depressive symptoms in adults [58] and adolescents [59]. Replacing sedentary behaviour with activity could influence depressive symptoms through various mechanisms, such as modulating neuroplasticity, reducing inflammation, and promoting self-esteem [60].

We also found novel evidence that potentially replacing sedentary behaviour with moderate-to-vigorous activity, or sleep was associated with a lower risk of anxiety symptom scores. However, replacing sedentary behaviour with light activity was associated with a higher risk of anxiety symptom scores. The finding is discordant with some previous studies that suggest broad increases in total physical activity volume may reduce the risk of anxiety disorders [22, 61]. The discrepancy could be due to the use of devices that can better estimate light activity than previous studies that have used self-report measures [62]. Light activity could capture restlessness, a common symptom of anxiety disorders that may contribute to this finding.

### Strengths and limitations

We took steps to reduce the risk of several sources of bias in this study. To the best of our knowledge, the UK Biobank is the world’s largest prospective cohort with accelerometer data. This sample size reduces some bias from random variability. The use of accelerometers that capture activity across the entire intensity spectrum, instead of self-report activity measures, should have lowered systematic bias due to measurement error. The use of e-values allowed us to assess the risk of bias from unmeasured confounding. We reduced the risk of reverse causation through a longitudinal design adjusting for symptoms at baseline and including a sensitivity analysis that excluded all participants with any history of depression or anxiety. We also used a compositional approach that allowed us to assess associations between sedentary behaviour and mental health while appropriately accounting for time spent in the rest of the day. The method also supports the estimation of replacement effects, which accounts for the co-dependent nature of time in different movement behaviours throughout the day and provides a more realistic representation of how reducing sedentary behaviour would occur. The a priori use of DAGs to inform our models, a comprehensive selection of variables available in the UK Biobank, and sensitivity analyses to explore alternative hypotheses improved our ability to estimate causal associations.

There were also several limitations to the study, including the possibility of selection bias. Only 5% of participants invited to join the UK Biobank were recruited, increasing the risk of selection bias in the sample. The full Biobank cohort is comparable to the general population across several sociodemographic and health factors [40, 63] but is healthier by other measures, such as smoking, obesity, or alcohol use [64]. The sample only includes middle-to-older-aged adults, and our results could only be generalisable to this demographic. Representativeness is a major issue for estimating prevalence, which was not the aim of our analysis. But there are also situations where participant selection and adjustments can induce collider bias, where two variables (e.g. exposure and outcome) can independently cause a third collider variable (e.g. participation in the study), and conditioning on the collider variable can distort associations between them [65]. Some recent studies have found representativeness issues in the UK Biobank can affect associations of lifestyle factors and mortality outcomes [66, 67].

The risk of bias by this mechanism is unclear as physical activity (our exposure) and depression and anxiety (our outcomes) estimates in the UK Biobank are similar to other nationally representative samples, such as the Health Survey for England and so appear unrelated to participation [63, 68, 69]. In our subsample, there were around 6.2% possible cases of depression and 3.7% for anxiety. These figures are comparable with nationally representative data suggesting a prevalence of 4.2% and 6.1% for depression and generalised anxiety disorders in middle-to-older adults from the Adult Psychiatric and Morbidity Survey [70]. However, the comparison of physical activity data uses self-reported measures as there is a lack of nationally representative device-based estimates of this data for the UK. In our subsample, the mean daily time in moderate-to-vigorous physical activity was 65.1 min, which is higher than accelerometer-based estimates from middle-aged adults in the 1970 British Cohort Study of 50.4 (men) and 51.6 (women) minutes per day [71]. However, direct comparisons are challenging as the cohort uses a thigh-worn accelerometer with different properties to the wrist-worn accelerometer in our sample.

The e-values indicate that the risk of unmeasured confounding nullifying our main findings is low, but it remains possible that several unmeasured confounding variables accumulate to have this effect. Despite our steps to reduce the risk of reverse causation, there could still be bias from measurement error of baseline symptoms. The baseline depression and anxiety symptoms measure also use a composite score, restricting our ability to estimate the incidence of possible cases for each condition at baseline. Sleep disturbances are also symptoms of depression and anxiety disorders, which may confound estimates of replacing sedentary behaviour with sleep. We only included measures of sleep duration, but sleep quality or the timing of sleep are other relevant factors to consider for mental health. The sleep variable was also from a self-reported measure, which may be subject to greater measurement error than the accelerometer data. However, gold-standard sleep measures are impractical for large-scale studies, such as direct observation and polysomnography, and self-reported sleep measures are common in other compositional studies [33, 35, 36]. Novel methods for estimating sleep using accelerometer data only are promising but still require further validation for large-scale studies [72, 73].

Advancing sedentary behaviour and physical activity research requires a shift to understanding behaviours within a 24-h cycle that includes sleep [32], and improved data collection methods suitable for large-scale research will continue to develop. While the use of accelerometers is a strength of this study, wrist-worn devices could misclassify some sedentary behaviours, such as standing [28]. Thigh or hip-worn devices are a useful option for assessing sedentary behaviour, but rare in large cohort studies. There is also mixed evidence as to whether a 7-day measurement of activity is representative of a typical week for most adults [74, 75]. People wearing accelerometers may increase their activity during the study period, which could underestimate the true association between activity and depression and anxiety symptoms here. Accelerometers also provide no contextual information about the movement that could modify its association with depression and anxiety symptoms. For example, time watching television may be differentially associated with mental health risks than working at a computer or reading [76, 77].

### Implications and future directions

Our findings suggest that reducing sedentary behaviour during the day could reduce the risk of depression and anxiety symptoms and disorders. However, interventions aiming to reduce sedentary behaviour must consider how different replacement activities might affect mental health. For example, we estimated that replacing sedentary behaviour with moderate-to-vigorous activity was associated with the lowest depression and anxiety symptom scores and could be a useful approach for interventions. Our results indicate that even small changes of less than an hour could be beneficial. For example, 15 or 30 min of brisk walking (moderate-intensity activity) per day could be sufficient to reduce mental health risks and a potentially more realistic target than 60-min changes in highly sedentary populations. Reducing sedentary behaviour with light activity could have a smaller effect on reducing depressive symptoms than moderate-to-vigorous activity but may be more acceptable and sustainable over long periods. Light activity may be easier to implement in daily routines and is typically more pleasurable, and yields greater motivation to engage than more intense activity [78].

Further studies using objective sleep measures are necessary to assess its possible influence relative to sedentary behaviour on mental health risks. Studies should also consider concurrent uses of devices and time-use diaries that provide additional contextual information about the activity to assess how replacing specific types of sedentary behaviours or at certain times in the day affects mental health risks. There is evidence that mentally passive sedentary behaviours (e.g. watching television) are associated with greater depression risks than mentally active sedentary behaviours (e.g. reading) [76, 77]. Replacing 60 min of watching television in the evening for sleep could have a greater impact on lowering the risk of depression than replacing 60 min of reading.

There is a lack of movement behaviour studies that assess anxiety symptoms despite their high prevalence and physical health risks [1]. Our finding that replacing sedentary behaviour with light activity was associated with higher anxiety symptoms highlights the need for more research. Future studies should consider how anxiety symptoms, such as restlessness, may produce micromovements that a wrist-worn accelerometer detects as light activity. The mechanisms underlying the relationship between activity and mental health could also differ for depression and anxiety and warrant a greater focus on studying anxiety symptoms. The finding could also reflect differences in the domain or type of physical activity, which can modify its association with mental health [79]. For example, light activity could more closely reflect time at work (e.g. moving around an office) or housework (e.g. washing dishes) that increase anxiety relative to moderate-to-vigorous activity that represents leisure time. Studies should incorporate these contextual factors to explore the nuances of these associations.

These findings also emphasise the nuances of understanding associations between movement and mental health in a 24-h context, where changing time in one behaviour inherently affects the time in another. Methodologies for studying behaviour within a 24-h time-use cycle will be necessary to advance the field of sedentary behaviour and physical activity research [32]. Prospective studies should utilise compositional approaches to account for time in other behaviours during the day appropriately and estimate replacement effects instead of only focusing on time in individual movement behaviours without appropriate adjustments. Further evidence from interventional studies will be useful in validating findings from these compositional studies.

## Conclusions

Our findings suggest that sedentary behaviour could be a risk factor for depression and anxiety disorders. More careful consideration of how best to replace sedentary behaviour is warranted. Replacing sedentary behaviour with moderate-to-vigorous activity could reduce the risk of depression and anxiety symptoms, including smaller replacements of less than 60 min. Replacing sedentary behaviour with light activity could be a sustainable and accessible approach for reducing the risk of depressive symptoms. However, whether this extends to anxiety symptoms and the extent to which sleep is beneficial over sedentary behaviour for depression and anxiety risk requires more work.

Advancing the field requires a greater uptake of methods that appropriately account for movement behaviours, including sleep, within a 24-h framework, such as compositional data analysis. There should also be a greater emphasis on assessing anxiety symptoms, which are highly prevalent but receive substantially less attention than depression.

## Supplementary Information

**Additional file 1: **Contains a flowchart of participants in the study (**Figure 1**), additional details on the exposure (**Methods 1**), a graph of our causal assumptions (**Figure 2**), additional details on compositional data analysis (**Methods 2**), the outcome distributions (**Figures 3 and 4**), base model results (**Table 1**), and sensitivity analyses results (**Results 1 and 2**).

## Data Availability

The corresponding author had full access to the data and takes responsibility for its integrity.

## Abbreviations

PHQ-9: Patient Health Questionnaire-9
GAD-7: Generalised Anxiety Disorder-7
DAG: Directed acyclic graph
95% CI: 95% confidence interval
OR: Odds ratio
