# Supplementary material for: Impact of replacing sedentary behaviour with other movement behaviours on depression and anxiety symptoms: a prospective cohort study in the UK Biobank
Source: BMC Med. 2021 Jun 17;19:133. doi: 10.1186/s12916-021-02007-3 (PMC8210357; doi:10.1186/s12916-021-02007-3)
Supplement: Supplementary file 1 — Additional file 1: Contains a flowchart of participants in the study (Figure 1), additional details on the exposure (Methods 1), a graph of our causal assumptions (Figure 2), additional details on compositional data analysis (Methods 2), the outcome distributions (Figures 3 and 4), base model results (Table 1), and sensitivity analyses results (Results 1 and 2). [file 12916_2021_2007_MOESM1_ESM.docx]

**Supplementary materials**

**Figure 1. Flow of participants included in this study**

Full UK Biobank sample (n = 502, 618)

Participants invited to wear accelerometers (n = 240,000)

Participants who provided accelerometer data (n = 103,706)

Participants with good quality accelerometer data and sufficient wear time (n = 99,608)

Participants with accelerometer data and a completed outcome (n = 60,235)

**Methods 1. Exposure: 24-hour movement behaviours**

From participants who provided a valid e-mail address at baseline, 236, 507 (47.1%) were invited to wear an accelerometer for seven days between February 2013 and December 2015. Researchers chose participant email addresses at random, except for those in the North West region to avoid overburdening participants who had already been recruited into trials for other new projects. A total of 103,706 participants (20.6%) agreed to wear the accelerometer, and 99,608 provided sufficient quality data for analysis (1). Participants wore an Axivity AX3 triaxial accelerometer on the wrist. The device performs similarly in estimating acceleration on multi-axis shake tests to the GENEActiv device used in other large, population-based cohort studies, including Whitehall II and Pelotas cohorts (2,3). The triaxial devices are validated with high precision in pre-living conditions for estimating activity and total energy expenditure, showing strong agreement and low population-level bias (~6%) compared with gold standard measures (doubly labelled water) in adults (4). After mailing the devices to participants, researchers initiated recording two days later at 10 am to continuously record activity at a sampling rate of 100Hz (1Hz is one sample per second) with a dynamic range of ±8*g* over seven consecutive days (1). Researchers asked participants to continue with normal daily activities during the recording period and always to wear the device, including while sleeping or bathing. Participants then returned the devices to a coordinating centre in pre-paid postage envelopes after the recording period.

Doherty *et al.* (1) processed the raw accelerometer data using protocols described in detail elsewhere (1). They calibrated raw accelerometer signals to local gravity (1*g*) to ensure consistent outputs from different devices under similar conditions and removed sensor noise. To derive movement intensity, they calculated Euclidean Norm Minus One (ENMO), by subtracting one gravitational unit (1g) from the vector magnitude of acceleration across three axes, the Euclidean norm. They defined non-wear time as periods where the standard deviation of all three axes was <13 milli-*g* for periods of ≥60 minutes and imputed non-wear time using data from similar time of day vector magnitudes and intensity distributions. Participants were excluded from the analysis if they had <72 hours of recording (n = 6,978) or poor-quality data (n = 120) to improve the reliability of estimates.

We followed protocols of previous studies (4–6) to define sedentary behaviour, light, and moderate-to-vigorous activity over 5-second epochs as averaged ENMO values of ≤30 milli-*g* (minus self-reported sleep duration), >30 milli-*g* and <125 milli-g and ≥125 milli-*g*, respectively. We derived the sleep duration variable from a touchscreen questionnaire that participants completed at baseline. The questionnaire asked: "About how many hours sleep do you get in every 24 hours? (please include naps)". The questionnaire automatically rejected responses of less than 1 or over 23 and asked participants to confirm responses of <3 or >12.

**
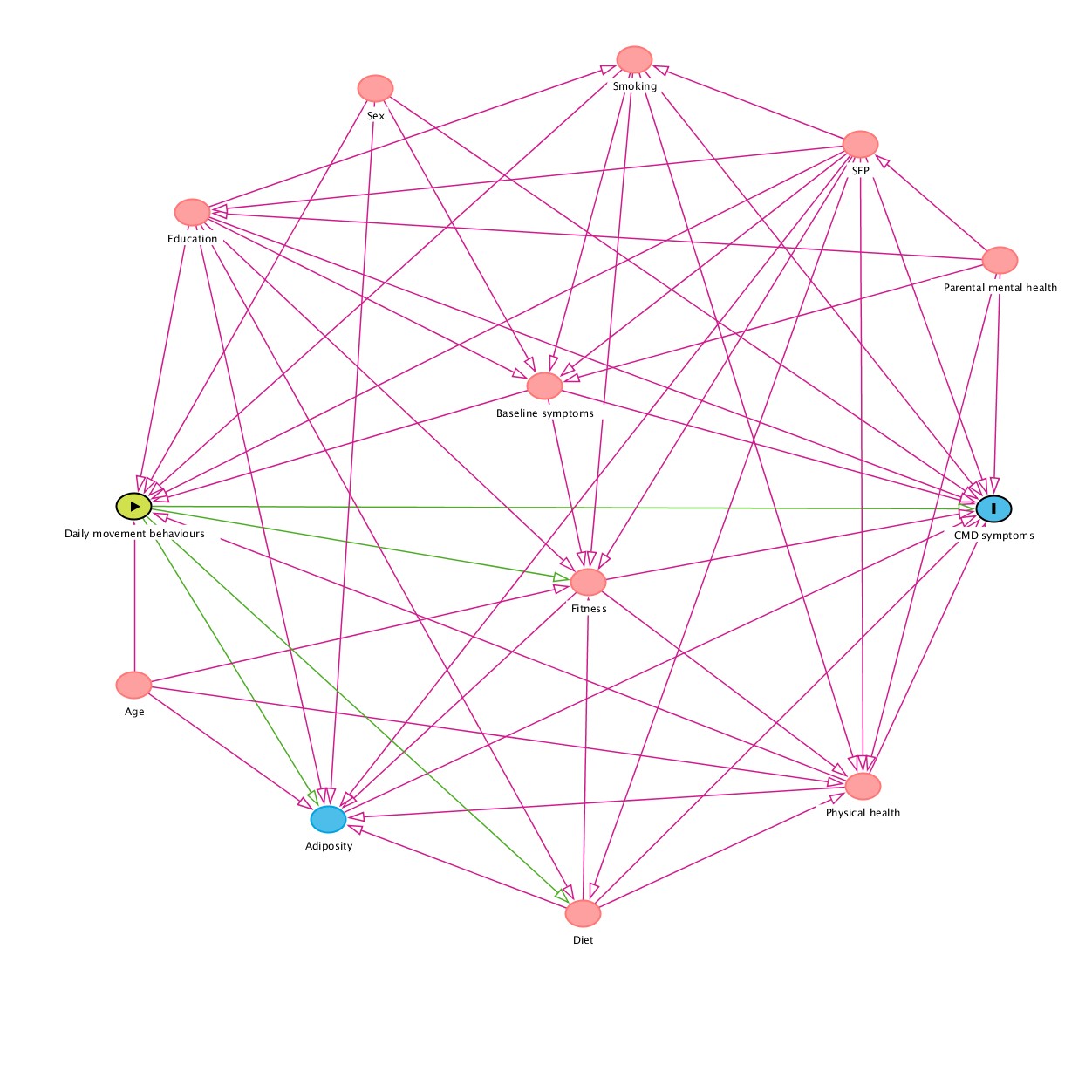
Figure 2. Directed acyclic graph of causal associations between covariates in this analysis**

*We used an online software package to generate this figure from Dagitty.net.*

*SEP = socioeconomic position, CMD symptoms = common mental health disorder (depression and anxiety) symptoms.*

**Methods 2. Compositional data analysis**

Compositional methods allow the inclusion of linearly independent variables representing time in sedentary behaviour, physical activity, and sleep over 24 hours, without inducing collinearity as in other approaches, such as standard multivariable regression. Standard approaches analyse data in real (Euclidean) space where variables are unconstrained, such as continuous regression lines representing different time-use variables. In this case, time-use variables contain absolute information about an activity without accounting for others. Compositional approaches use log transformations to move data into a geometric (Cartesian) space, where all lines representing a time-use variable are constrained by each other. This space is a ‘simplex’, where time in sedentary behaviour, physical activity, and sleep are co-dependent. We calculate log-ratio coordinates for each participant to represent each time-use variable and contain relative information, such as daily sedentary behaviour relative to physical activity and sleep. The term coordinate refers to the locations in the simplex, such that it is a particular composition of sedentary behaviour, physical activity, and sleep within a sample space of all possible variations of the composition. The coordinates essentially map compositions from Cartesian space (a simplex) to Euclidean space, which means we can enter them as exposure variables into a standard regression model while maintaining their relative information. The relative information contained within each composition means that any changes in sedentary behaviour would account for physical activity and sleep.

Each exposure of interest in our analysis is a composition of sleep, sedentary behaviour, light, and moderate-to-vigorous activity, with a mean of daily time adding up to 1440 minutes (24-hours). We used a pivot coordinate approach whereby we calculated a set of three isometric log-ratio coordinates per participant that represents their total relative time in each movement behaviour per day. The first (pivot) coordinate represents daily sedentary relative to the geometric mean of all other daily movement behaviours, i.e., sleep, light, and moderate-to-vigorous activity. The other two log-ratio coordinates contain relative information representing the remaining time in a participant’s total daily composition, i.e., sleep over light and moderate-to-vigorous, and light over moderate-to-vigorous activity.

Each participant has three isometric log-ratios that represents their average daily time-use composition, which we use as the exposure variable in our analysis. After calculating the base models, we use a change-matrix procedure to estimate replacement effects, described in detail elsewhere (7). The procedure involves inverse log-transforming the base model coefficients to obtain the composition associated with them in the simplex. Applying the change-matrix procedure to these coefficients (8) can estimate changes in depression or anxiety scores associated with a changes in the composition of sedentary behaviour, sleep, light, and moderate-to-vigorous activity. We simulated estimated mean changes in depression and anxiety scores in response to unit changes of 1 to 60 minutes of daily sedentary behaviour with the equivalent replacements for each movement behaviour.
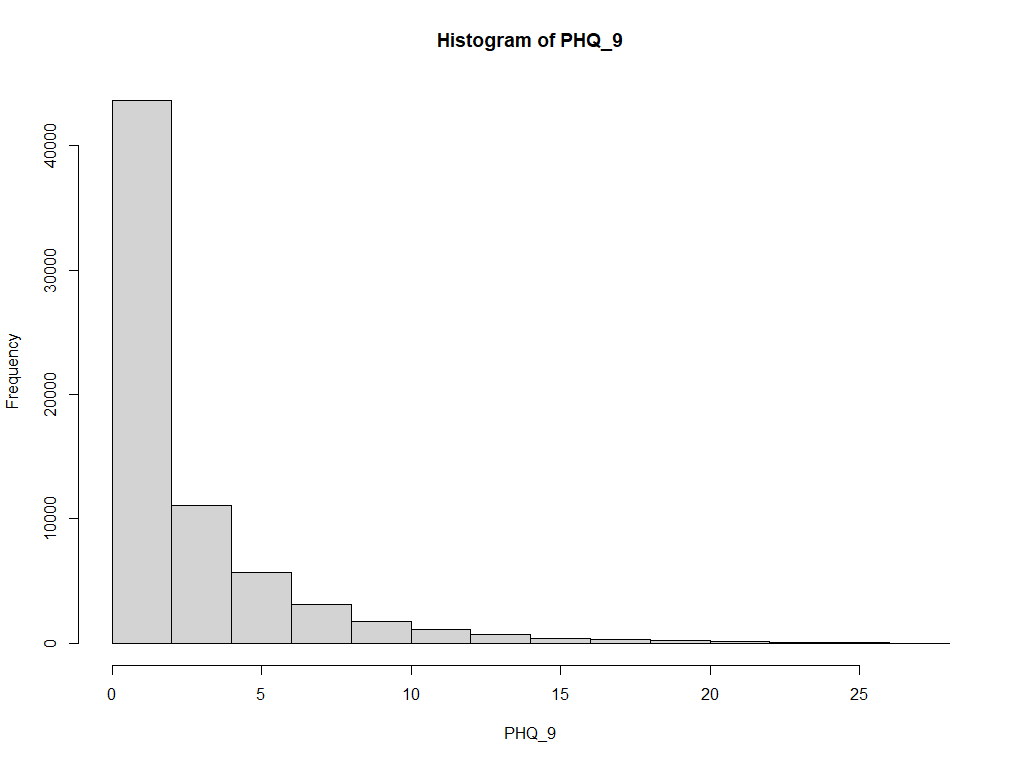
**Figure 3. PHQ-9 score distribution**

*Residual deviance: 267776 on 68369 degrees of freedom, indicating overdispersion.*


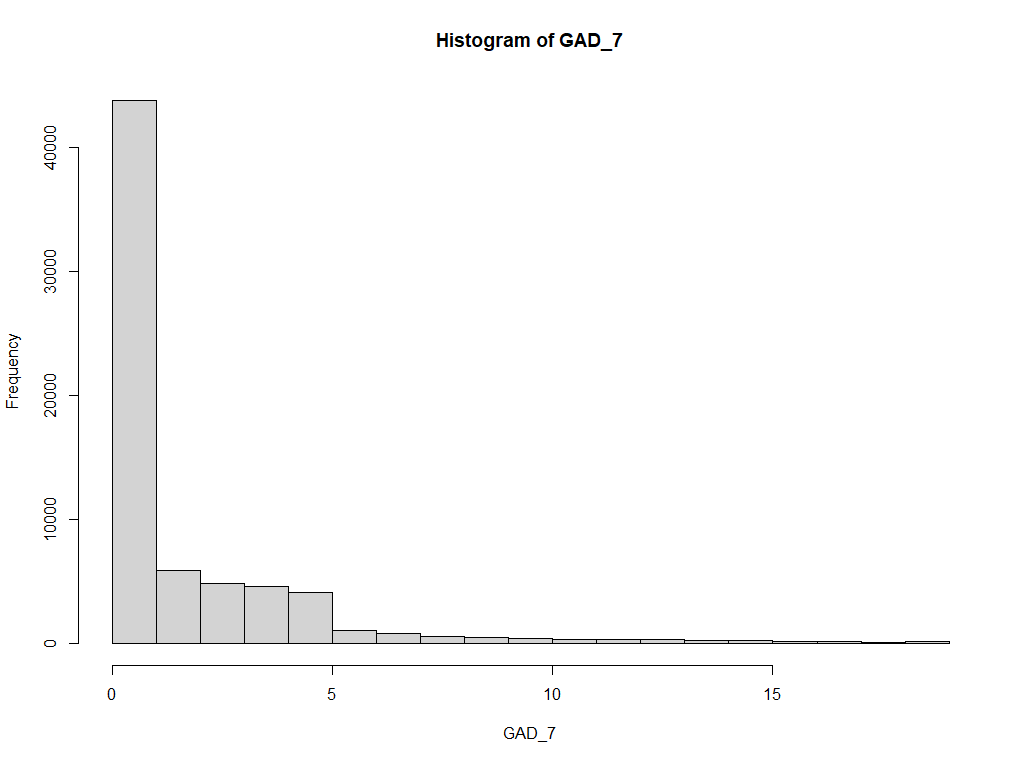
**Figure 4. GAD-7 score distribution**

*Residual deviance:252997 on 68350 degrees of freedom, indicating overdispersion.*

|  |  | Depression (PHQ-9) | | | | Anxiety (GAD-7) | | | |
| --- | --- | --- | --- | --- | --- | --- | --- | --- | --- |
| Model | Log-ratio coordinates* | exp($\gamma$)** | Lower confidence interval | Upper confidence interval | P value | exp($\gamma$)** | Lower confidence interval | Upper confidence interval | P value |
| Unadjusted | $z_{1}$ | 0.578 | 0.530 | 0.625 | <0.001 | 0.517 | 0.453 | 0.581 | <0.001 |
|  | $z_{2}$ | 0.247 | 0.204 | 0.290 | <0.001 | -0.061 | -0.118 | -0.004 | <0.001 |
|  | $z_{3}$ | 0.207 | 0.172 | 0.242 | <0.001 | 0.254 | 0.207 | 0.302 | <0.001 |
| Adjusted | $z_{1}$ | 0.491 | 0.442 | 0.539 | <0.001 | 0.373 | 0.305 | 0.440 | <0.001 |
|  | $z_{2}$ | 0.165 | 0.121 | 0.208 | <0.001 | -0.139 | -0.199 | -0.079 | <0.001 |
|  | $z_{3}$ | 0.135 | 0.099 | 0.172 | <0.001 | 0.136 | 0.086 | 0.187 | <0.001 |

**Table 1. Estimates for negative binomial regression base model with depression and anxiety outcomes**

**Log ratio coordinates representing*

${(z}_{1},z_{2}{,z}_{3})=\left( \sqrt{\frac{3}{4}}\ln\frac{sedentary behaviour}{{(light \cdot moderate-to-vigorous \cdot sleep)}^{1/3}},\sqrt{\frac{2}{3}}\ln\frac{light}{{( moderate-to-vigorous \cdot sleep)}^{1/2}},\sqrt{\frac{1}{2}}\ln\frac{moderate-to-vigorous}{sleep} \right)$

*In this model, only z1 is interpretable as it contains relative information for all 24h behaviours (i.e., sedentary behaviour, light and moderate-to-vigorous physical activity, and sleep). The p values indicate a statistically significant association between sedentary behaviour (z1) and depression and anxiety, after account for the time spent in the rest of the 24h movement behaviours. The direction of the coefficient indicates a positive association for both outcomes. In compositional methods, it is not possible to interpret the coefficient as the strength of association as in typical linear regression as the coefficients purposely represent ratios, not absolute values* (7)*. For this reason, we did not back-transform these coefficients in this base model as we do for subsequent models where we estimate replacement effects in the main analysis, which require clearly interpretable units of change.*

***Exponential of regression coefficient (*$\gamma$*). This represents the unit change in depression or anxiety scores per unit increase in each log-ratio of the time allocated to the behaviour in the numerator against the geometric mean of the others in the denominator.*

**Results 1. Models after excluding people with a history of depression or anxiety**

In this sample, substituting 60 minutes of sedentary behaviour for light, moderate-to-vigorous activity, and sleep was associated with 1.1% (95% CI 0.1% to 2.1%), 13.7% (95% CI 12.4% to 15.0%), and 9.6% (95% CI 8.7% to 10.6%) lower depression scores. Replacing 60 minutes of sedentary behaviour with moderate-to-vigorous activity and sleep was associated with 7.7% (95% CI 6.5% to 9.0%), and 5.9% (95% CI 5.0% to 6.7%) lower anxiety scores and 5.4% (95% CI 4.4% to 6.3%) higher scores with light activity.

**Results 2. E-values**

The e-values estimates the required strength of an unmeasured confounding variable to nullify the observed associations between our exposure and outcomes. For depression scores, the e-values as incident rate ratios for replacing 60 minutes of sedentary behaviour with light, moderate-to-vigorous activity, and sleep were 1.13 (CI = 1.07), 1.55 (CI = 1.51), and 1.38 (CI = 1.36). For anxiety scores, the e-values for replacing 60 minutes of sedentary behaviour with light, moderate-to-vigorous activity, and sleep were 1.26 (CI = 1.23), 1.35 (CI = 1.31), and 1.27 (CI = 1.24).

**References**

1. Doherty A, Jackson D, Hammerla N, Plötz T, Olivier P, Granat MH, et al. Large Scale Population Assessment of Physical Activity Using Wrist Worn Accelerometers: The UK Biobank Study. Buchowski M, editor. PLoS One [Internet]. 2017 Feb 1;12(2):e0169649.

2. Da silva ICM, Van hees VT, Ramires V V., Knuth AG, Bielemann RM, Ekelund U, et al. Physical activity levels in three Brazilian birth cohorts as assessed with raw triaxial wrist accelerometry. Int J Epidemiol [Internet]. 2014 Dec 1;43(6):1959–68.

3. Sabia S, Van Hees VT, Shipley MJ, Trenell MI, Hagger-Johnson G, Elbaz A, et al. Association between questionnaire-and accelerometer-assessed physical activity: The role of sociodemographic factors. Am J Epidemiol [Internet]. 2014;179(6):781–90.

4. White T, Westgate K, Hollidge S, Venables M, Olivier P, Wareham N, et al. Estimating energy expenditure from wrist and thigh accelerometry in free-living adults: a doubly labelled water study. Int J Obes [Internet]. 2019 Nov 1;43(11):2333–42.

5. Kim Y, White T, Wijndaele K, Sharp SJ, Wareham NJ, Brage S. Adiposity and grip strength as long-Term predictors of objectively measured physical activity in 93 015 adults: The UK Biobank study. Int J Obes [Internet]. 2017 Sep 1;41(9):1361–8.

6. Hajna S, White T, Panter J, Brage S, Wijndaele K, Woodcock J, et al. Driving status, travel modes and accelerometer-assessed physical activity in younger, middle-aged and older adults: A prospective study of 90 810 UK Biobank participants. Int J Epidemiol. 2019;48(4):1175–86.

7. Chastin SFM, Palarea-Albaladejo J, Dontje ML, Skelton DA. Combined effects of time spent in physical activity, sedentary behaviors and sleep on obesity and cardio-metabolic health markers: A novel compositional data analysis approach. PLoS One. 2015;10(10).

8. Palarea-Albaladejo J, Martín-Fernández JA. ZCompositions - R package for multivariate imputation of left-censored data under a compositional approach. Chemom Intell Lab Syst. 2015 Apr 5;143:85–96.
